# Winter Wheat Yield Response to Plant Density as a Function of Yield Environment and Tillering Potential: A Review and Field Studies

**DOI:** 10.3389/fpls.2020.00054

**Published:** 2020-03-05

**Authors:** Leonardo M. Bastos, Walter Carciochi, Romulo P. Lollato, Brent R. Jaenisch, Caio R. Rezende, Rai Schwalbert, P.V. Vara Prasad, Guorong Zhang, Allan K. Fritz, Chris Foster, Yancy Wright, Steven Young, Pauley Bradley, Ignacio A. Ciampitti

**Affiliations:** ^1^ Department of Agronomy, Kansas State University, Manhattan, KS, United States; ^2^ John Deere, Johnston, IA, United States

**Keywords:** wheat, yield environment, tillering potential, yield components, synthesis-analysis

## Abstract

Wheat (*Triticum aestivum* L.) grain yield response to plant density is inconsistent, and the mechanisms driving this response are unclear. A better understanding of the factors governing this relationship could improve plant density recommendations according to specific environmental and genetics characteristics. Therefore, the aims of this paper were to: i) execute a synthesis-analysis of existing literature related to yield-plant density relationship to provide an indication of the need for different agronomic optimum plant density (AOPD) in different yield environments (YEs), and ii) explore a data set of field research studies conducted in Kansas (USA) on yield response to plant density to determine the AOPD at different YEs, evaluate the effect of tillering potential (TP) on the AOPD, and explain changes in AOPD *via* variations in wheat yield components. Major findings of this study are: i) the synthesis-analysis portrayed new insights of differences in AOPD at varying YEs, reducing the AOPD as the attainable yield increases (with AOPD moving from 397 pl m^-2^ for the low YE to 191 pl m^-2^ for the high YE); ii) the field dataset confirmed the trend observed in the synthesis-analysis but expanded on the physiological mechanisms underpinning the yield response to plant density for wheat, mainly highlighting the following points: a) high TP reduces the AOPD mainly in high and low YEs, b) at canopy-scale, both final number of heads and kernels per square meter were the main factors improving yield response to plant density under high TP, c) under varying YEs, at per-plant-scale, a compensation between heads per plant and kernels per head was the main factor contributing to yield with different TP.

## Introduction

Wheat is one of the most important cereals in human diets, increasing its relevancy as the global population is projected to increase by 30% in 2050 (United Nations, 2019). Thus, a continuous increase in wheat demand is expected, which will be mainly satisfied by improving crop yield per unit area (Neumann et al., 2010) as expansion in cultivated land is unlikely due to negative social and environmental impacts (Foley et al., 2011). At a global scale, the wheat yield gap (deviation of actual from potential yield) was estimated at 36% (Neumann et al., 2010), but this yield gap is much larger in regions such as the U.S. southern Great Plains (Patrignani et al., 2014; Lollato et al., 2017). Among the most relevant management factors for improving wheat yields and closing the yield gap is the use of the optimum seeding rate for an appropriate plant density (PD) (Hochman and Horan, 2018; Jaenisch et al., 2019; Lollato et al., 2019).

Below-optimum seeding rates may reduce resource use efficiency, yield, and final profit (Whaley et al., 2000), depending on the level of resource availability (Lollato et al., 2019; Fischer et al., 2019). Meanwhile, above-optimum seeding rates increase cost of production and might potentially decrease yield by increasing disease pressure, insects, and lodging (Lloveras et al., 2004; Laghari et al., 2011). Consequently, defining the agronomic optimum plant density (AOPD), which is the minimum number of plants per unit area required to maximize yield, is crucial for future improvements in wheat yield. Nonetheless, one of the main challenges of determining the AOPD is that diverse yield to PD relationships have been reported in the scientific literature for wheat, which range from linear, quadratic, quadratic-plateau, and lack of response (Whaley et al., 2000; Lloveras et al., 2004; Fischer et al., 2019). Thus, it is necessary to understand yield to PD response models *via* synthesizing studies published in the scientific literature and analyzing a comprehensive field research data set.

The interplay between genotype × environment × management (G × E × M) regulates wheat plasticity and attainable yield (Geleta et al., 2002; Valério et al., 2013), rendering AOPD dependent on yield environment (YE). In this line, recent studies in soybean [*Glycine max*(L.) Merr.] (Corassa et al., 2018; Carciochi et al., 2019), canola (*Brassica napus* L. “Canola”) (Assefa et al., 2018b), and maize (*Zea mays* L.) (Assefa et al., 2016; Assefa et al., 2018a) classified the data on different YE levels based on its average yield and determined the AOPD at each YE. Therefore, as was observed for canola and soybean (e.g., crops that have compensation mechanisms comparable to wheat), the AOPD in wheat could change across YEs with a greater requirement of plants to attain the maximum yield at the low YE. However, this hypothesis is yet to be tested.

Wheat yield components have a strong compensation capacity depending on the availability of resources (Whaley et al., 2000; Lloveras et al., 2004). However, this compensatory mechanism could differ across wheat genotypes (Dahlke et al., 1993; Lloveras et al., 2004). As an example, some wheat genotypes have greater tillering potential (TP) than others (Valério et al., 2013), so if the number of plants is below the carrying capacity of resources available, the number of tillers might increase to compensate the lack of plants. Thus, it is possible that the AOPD could depend on the genotype's TP within each YE. Other yield components such as kernel number and weight are modified with changes in the PD. Thus, increases in PD usually increase heads and kernel number per unit area, and decrease kernel weight and kernels per head (Geleta et al., 2002; Lloveras et al., 2004; Valério et al., 2013). However, the magnitude of these changes could depend on the availability of resources at each YE, so the variation in yield components at different YEs deserves to be studied.

The overarching objective of this study was to quantify whether the AOPD for winter wheat depended on YE and genotype TP. We used two levels of organization based on the type of data utilized in the analyses to attain this goal: i) a synthesis-analysis of existing data related to yield-PD relationship with the specific objective of providing an indication of the need for different AOPD at each YE, and ii) an analysis of a comprehensive dataset of field research studies on winter wheat yield to PD to determine the AOPD at different YEs, evaluate the effect of TP on the AOPD, and explain changes in AOPD *via* modifications in wheat yield components.

## Material and Methods

### Synthesis-Analysis

A literature review was conducted to retrieve data from published scientific research using Web of Science™ and Google Scholar. The criteria for inclusion of a paper in the database were: i) the study must have been performed with winter wheat (i.e., no spring wheat or durum wheat (*Triticum durum* L.) studies were included); ii) the study must have reported both PD (i.e., measured final number of plants per area, not only reporting seeding rates) and yield, and iii) the study must have been conducted in North America [United States (US) and Canada] and published during the period from 1980 to 2019. From all the papers screened in the research literature (**Table 1**), a database containing information on citation (author, year of publication), location, site-years, number of observations reported, average reported PD and yield (minimum and maximum) were recorded from each study. Whenever grain yield and PD were reported in figure format, data were extracted using the WebPlotDigitizer software version 4.2.

**Table 1:** Characterization of studies included in the synthesis analysis. Location is shown as state/province initial followed by country initial.

Citation	No. Sites, location	Pest control (W/I/D)	Site- years	Obs.	Plant density	Yield
					(plants m^-2^)	(Mg ha^-1^)
Joseph et al. (1985)	1, VA/USA	Y/Y/Y	3	24	364 (148, 872)	6.7 (5, 8.1)
McLeod et al. (1992)	2, SK/CAN	Y/Y/Y	7	20	120 (70, 170)	1.2 (0.6, 1.9)
Mian and Nafziger (1992)	1, IL/USA	NA	2	6	169 (121, 250)	3.6 (2.9, 4.5)
McLeod et al. (1996)	2, SK/CAN	Y/Y/NA	6	22	97 (54, 190)	1.3 (0.3, 2.6)
Roberts et al. (2001)	2, OK/USA	Y/NA/NA	2	6	299 (186, 444)	2.1 (1.6, 2.7)
Schaafsma and Tamburic-Ilincic (2005)	1, ON/CAN	NA/NA/Y*	2	3	613 (343, 832)	2.1 (2, 2.3)
McKenzie et al. (2007)	3, AB/CAN	Y/NA/NA	9	5	191 (154, 238)	6.4 (6.2, 6.4)
Childress et al. (2010)	5, VA/USA	NA/NA/Y	6	5	355 (237, 495)	4.6 (4.4, 4.7)
Beres et al. (2016)	9, AB, MB, SK/CAN	Y/Y*/Y*	26	22	153 (40, 340)	4.3 (1.6, 8.1)
Bhatta et al. (2017)	1, NE/USA	NA	2	6	233 (122, 365)	3.9 (2.1, 5.7)

*As part of the study treatment design.

USA, United States of America; CAN, Canada; VA, Virginia; SK, Saskatchewan; IL, Illinois; OK, Oklahoma; ON, Ontario; AB, Alberta; MB, Manitoba; NE, Nebraska; W, weeds; I, insects; D, diseases; Y, yes; NA, not available.

Plant density and yield are shown as median (minimum, maximum).

To evaluate the response of winter wheat grain yield to PD across all studies, four different models were fit to the 1^st^, 50^th^, and 99^th^ quantiles of the dataset using the functions *rq* and *nlrq*, for linear and non-linear regression respectively, from the package *quantreg* (Koenker, 2019) in R (R Core Team, 2019). These quantiles were chosen to represent low, medium, and high yielding conditions, respectively. Models tested were the linear, quadratic, linear-plateau and quadratic-plateau. For each quantile, the model with the lowest Akaike Information Criteria (AIC) was used to estimate AOPD.

### Field Research Studies

Nine field experiments resulting from the combination of sites and years were conducted during the winter wheat growing seasons of 2015-16, 2016-17, and 2017-18 in Kansas, USA. All experiments were sown at the optimum sowing window for each location to avoid the confounding and interacting effects of sowing date and wheat seeding rate (Staggenborg et al., 2003). Treatment structure was a two-way complete factorial combination of seeding rate by winter wheat genotype, and trials were established in a randomized complete block design with four blocks. Each field experiment consisted of five to seven winter wheat genotypes sown at five target seeding rates (150, 235, 321, 408, 494 seeds m^-2^) (**Table 2**). Five commercial genotypes were consistent across all experiments (i.e., “Joe”, “KanMark”, “Larry”, “Tanaka” and “Zenda”), and the other two commercial genotypes, when applicable, varied with site-year and included “1863”, “Ag Icon, “Bob Dole”, “Everest”, and “AM Cartwright”. Each experimental unit was 10 m long by seven rows (0.19-m spaced) except for two site-years (Hutchinson 2015–2016 and Hays 2017–2018) that consisted of six rows (0.25-m spaced).

**Table 2:** Geographic coordinates, yield environment classification (YE), soil type, tillage practice (CT, conventional till; NT, no-till), genotypes, and sowing date (MM/DD/YYYY) for each location and winter wheat growing season evaluated in the nine field studies.

Havrest Year	Location	YE	Latitude (°)	Longitude (°)	Soil Type	Tillage	Genotypes	Sowing date
2016	Manhattan	Low	39.218	-96.591	Kahola silt loam (Fine-silty, mixed, superactive, mesic Cumulic Hapludolls)	NT	Everest, KanMark, 1863, Zenda, Larry, Tatanka, Joe	10/08/2015
2016	Hutchinson	Medium	37.931	-98.027	Ost loam (Fine-loamy, mixed, superactive, mesic Udic Argiustolls)	CT	Everest, KanMark, 1863, Zenda, Larry, Tatanka, Joe	10/07/2015
2017		High					KanMark, Zenda, Larry, Joe, Tatanka, Ag Icon, Bob Dole	10/13/2016	
2018		Medium					KanMark, Zenda, Larry, Joe, Tatanka, Bob Dole, AM Cartwright	10/19/2017
2017	Belleville	Medium	39.815	-97.672	Crete silt loam (Fine, smectitic, mesic Pachic Udertic Argiustolls)	CT	KanMark, Zenda, Larry, Joe, Tatanka, Ag Icon, Bob Dole	10/03/2016
2018	Ashland Bottoms	Low	39.127	-96.635	Wymore silty clay loam (Fine, smectitic, mesic Aquertic Argiudolls)	CT	KanMark, Zenda, Larry, Joe, Tatanka, Bob Dole, AM Cartwright	10/06/2017
2018	Great Bend	High	38.364	-98.867	Taver loam (Fine, smectitic, mesic Udertic Argiustolls)	CT	KanMark, Zenda, Larry, Joe, Tatanka.	10/12/2017
2018	Leoti	Medium	38.285	-101.211	Richfield silt loam (Fine, smectitic, mesic Aridic Argiustolls)	NT	KanMark, Zenda, Larry, Joe, Tatanka	10/13/2017
2018	Hays	Low	38.856	-99.338	Harney silt loam (Fine, smectitic, mesic Typic Argiustolls)	CT	KanMark, Zenda, Larry, Joe, Tatanka, Bob Dole, AM Cartwright	10/03/2017

While row spacing and tillage practices varied with site-year (**Table 2**), crop husbandry was otherwise consistent across locations. Weeds were controlled during the fall prior to sowing and early in the spring using commercially available herbicides. Composite soil samples consisting of 15 individual soil cores were collected prior to sowing at the 0–0.15 and 0.15–0.6 m soil depth to characterize initial soil fertility and adjust N fertilizer rates (**Supplementary Table 1**). Soil analysis consisted of pH, buffer pH, ammonium, nitrate, Mehlich III P, K, Ca, Mg, Na, organic matter, cation exchange capacity, Cl, and sulfate-sulfur (Nathan and Gelderman, 2012). Diammonium phosphate (18-46-0) was applied in-furrow at sowing at a rate of 55 kg ha^-1^. Nitrogen fertilizer was applied as urea (46-0-0) during early spring in rates sufficient to meet a yield goal of 4.7 Mg ha^-1^ following Kansas State University's recommendations that considered nitrate-N available in the 0–0.6 m profile, N derived from mineralization of organic matter in the 0–0.15 m, previous crop, and tillage practices (Leikam et al., 2003). Foliar fungicide (i.e., 85 g ha^–1^ as Picoxystrobin-Class 11 plus 34 g ha^–1^ as Cyproconazole-Class 3) and a non-ionic surfactant (1905 g ha^–1^) was sprayed at heading (Zadoks GS 55) using a backpack sprayer with a CO_2_ tank and a hand boom. The use of fungicide was justified to avoid the confounding effects of genetic resistance to different fungal diseases. Daily weather data was collected for each site year from both Climate Engine (Huntington et al., 2017) and monitoring stations from Kansas Mesonet (http://mesonet.k-state.edu/), including maximum and minimum daily temperature, precipitation, evapotranspiration, and incident solar radiation (**Supplementary Table 2**).

### Measurements

Crop stand establishment was measured from one linear meter in two different places within each experimental unit approximately 20 to 30 days after sowing, and final stand on an area basis (plants m^-2^) was calculated considering row spacing in each location. While we did not measure the stand after the winter to quantify winterkill, the studied growing seasons were not conducive to winterkill due to smooth transitions to colder temperatures, allowing the crop to acclimate and improve freeze tolerance (Bridger et al., 1994). Since the relationship between achieved and target plant densities can vary between YEs and serve as a feedback to the overall observed yield level, we evaluated the achieved/target stand ratio within each YE. At physiological maturity (Zadoks GS94), shoot biomass samples were collected from a linear meter per experimental unit. Samples were dried in an air-forced dryer at 60 °C for approximately one week. These samples were used to measure the yield components: i) shoot biomass, ii) harvest index (grain yield to total aboveground biomass ratio), iii) heads per linear meter (later transformed into heads m^-2^ using the row spacing), iv) kernels head^-1^, and v) thousand-kernel weight. Grain yield was measured by combine-harvesting the entire experimental unit. Grain moisture content was measured at harvest time and yields corrected for 130 g kg^-1^ moisture content.

### Statistical Analysis

Different site-years were grouped into low, medium, and high YEs (**Table 2**) using a fuzzy k-means clustering algorithm across all raw grain yield data points (*n =* 1160), implemented with the function *fanny* (utilizing Euclidean distance as the dissimilarity metric) from the package *cluster* (Maechler et al., 2019) in R. The final number of groups (i.e. three) was chosen because it most parsimoniously minimized intra-group variance while maximizing inter-group variance. Final site-year association into a YE group was based on the majority (> 50%) YE membership of data points within a given site-year. The generated YEs are groups that represent a simplification of site-specific characteristics impacting grain yield.

Different genotypes were grouped as having low or high TPs. For that, first the complete dataset was filtered to include only the target seeding rate treatment level of 148 seeds m^-2^ (i.e., the lowest seeding rate which resulted in an average density of 133 plants m^-2^ and varied from 57 to 335, and therefore under which TP is most expressed). This first step ensured that the TP of the different varieties could be expressed due to the low PD. Then, the average number of heads per plant was calculated for each genotype as the number of heads m^-2^ measured in the stand count [c.a., 20 to 30 days after sowing (Mehring, 2016)], and this was used in a fuzzy k-means clustering algorithm in a similar manner as previously described to segregate high- versus low TP genotypes. This methodology expanded on the current literature to classify varieties in high and low TP (e.g., Mehring, 2016) by using a clustering approach and by only evaluating tiller production in extremely low PD to allow for TP expression (e.g., Kuraparthy et al., 2007).

Daily weather data variables were summarized (summed or averaged) for each YE for the periods of fall (October through November), winter (December through February), jointing through anthesis (March through April), and grain filling (May through mid-June). To assess differences in weather conditions between YEs, an analysis of variance (ANOVA) was conducted for each weather variable with the explanatory variables of YE, period, and their interaction using the function *lm* from the package *stats* (R Core Team, 2019) in R. Terms in the ANOVA were deemed significant at α = 0.05.

The AOPD for each YE × TP combination was estimated by choosing the AIC-based best-fit model describing the relationship between grain yield and PD. Models tested were the intercept-only, linear, quadratic, and linear-plateau. For the intercept-only, linear, and quadratic models, the function *lmer* from the package *lme4* (Bates et al., 2015) in R was used to include site-year as a random effect either alone (intercept-only) or in addition to PD as a fixed effect variable (linear and quadratic models). For the linear-plateau model, the function *nlme* from the package *nlme* (Pinheiro et al., 2019) in R was used to include site-year as a random effect in addition to PD as a fixed effect variable in a non-linear shape of the form

GY=a+b×PD (if PD<tx)

where GY is grain yield (Mg ha^-1^); PD is plant density (plants m^-2^); and the coefficients a (y-intercept), b (slope), and tx (breakpoint projected on PD).

To dissect the differential yield responses to PD, winter wheat yield and its components of heads per plant, heads m^-2^, kernels head^-1^, kernels m^-2^, and thousand-kernel weight were analyzed as a function of PD group, YE, and TP. For that, PD was grouped into discrete intervals of <100, 100–200, 200–300, 300–400, and >400 plants m^-2^. A mixed-effect ANOVA model was fit with each of yield or yield components as the response variable, and the explanatory variables of YE, TP, PD, and their interactions as fixed effect terms, and block nested in site-year as random effect. Significant (α = 0.05) ANOVA terms were further analyzed by conducting pairwise comparisons of the expected marginal means using Fisher's least significant difference test.

The overall importance of yield components in explaining grain yield variability was assessed. For that, three different random-effect models were fit where the response variable grain yield was regressed against different sets of yield component as random effects, using the function *lmer* from the package *lme4* (Bates et al., 2015) in R. The first model represented an orthogonal partition of yield components and included PD, heads per plant, kernels head^-1^, and thousand-kernel weight; the second model represented the main yield components defining final grain yield and included kernels m^-2^ and thousand-kernel weight; and the third model represented the contribution of harvest index and aboveground biomass to total grain yield variance.

To understand how different yield components were affected by YE and TP at AOPD, the dataset was further filtered to include only observations within ± 50 plants m^-2^ of the estimated YE-TP-specific AOPD, except for high-YE high-TP. For the latter, AOPD was derived from the intercept-only model and estimated at the minimum PD, filtering to include observations within AOPD+100 plants m^-2^. Thereafter, a mixed-effect ANOVA model was fit for each of yield or yield components as the response variable, and the explanatory variables of YE, TP, and their interactions as fixed effect terms, and site-year as random effect term. Terms in the ANOVA were deemed significant at α = 0.05. Significant terms were further analyzed by conducting pairwise comparisons of the expected marginal means using Fisher's least significant difference test.

## Results

### Winter Wheat Yield Response to Plant Density in North America

**Figure 1:**
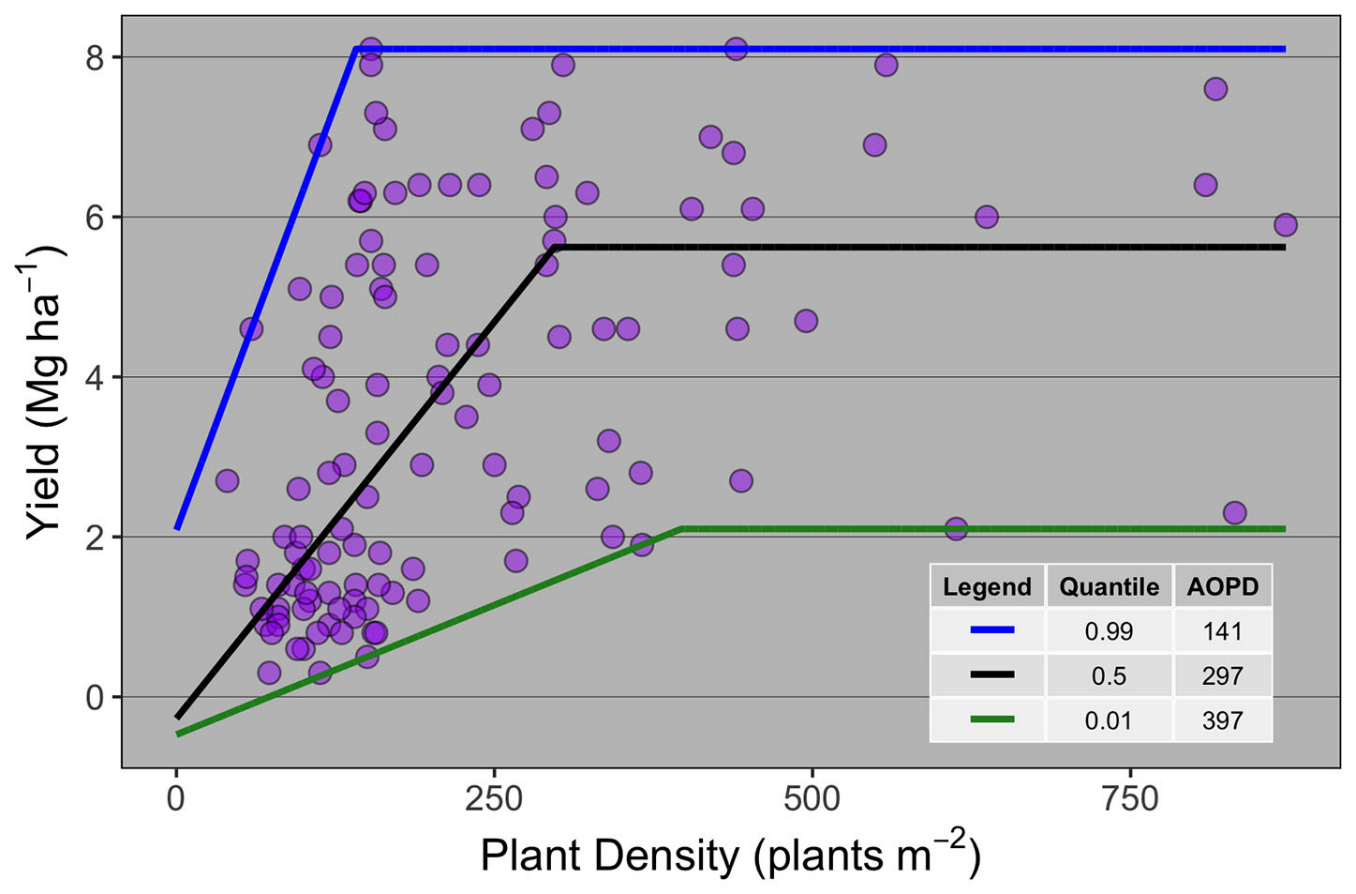
Winter wheat grain yield response to plant density for data collected from 10 publications (*n =* 119). Each point represents the average yield at a given plant density as reported on the publication from which it was extracted. Different lines are the best-fit curve describing the agronomic optimum plant density (AOPD) at the 1^st^, 50^th^, and 99^th^ quantiles.

Ten publications with field studies conducted in North America were found in the literature reporting both winter wheat yield and PD (**Table 1**), for a total of 119 observations. Despite a relatively small number of observations, there was a large range in PD (40 to 872 plants m^-2^) and in grain yield (0.3 to 8.1 Mg ha^-1^) among the studies matching our inclusion criteria. Linear-plateau models had the best fit for the 1^st^, 50^th^, and 99^th^ quantiles (**Figure 1**), and AOPD was estimated at 397, 297, and 141 plants m^-2^ for the low, medium, and high YE, respectively. Likewise, the slope of the linear phase differed among YE, suggesting that each additional emerged plant per m^2^ produced more yield in the high YE (0.043 Mg ha^-1^.plant m^-2^) versus the medium or low YE (0.020 and 0.006 Mg ha^-1^.plant m^-2^). Of the ten studies included in the synthesis analysis, only three stated that weeds, insects, and diseases were controlled, two studies did not mention pest control of any sort, while most of the other studies mentioned control of only one or two pest types (**Table 1**).

### Winter Wheat Yield as a Function of G × E × M

**Figure 2:**
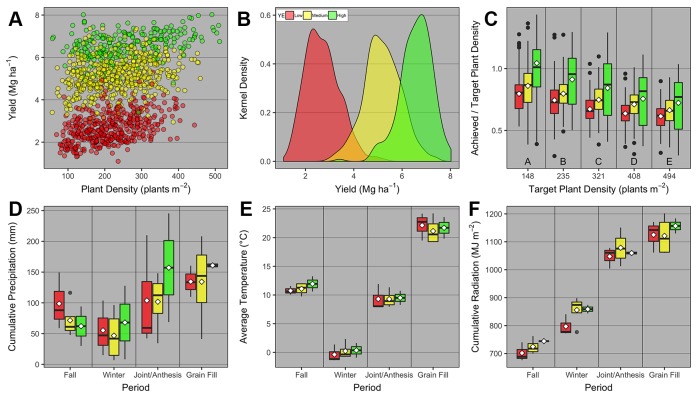
Relationship between **(A)** winter wheat grain yield and plant density for low, medium, and high yield environments (YE); **(B)** kernel density distribution for grain yield at each YE; **(C)** achieved and target plant density ratio vs. target plant density for each YE; boxplots of **(D)** cumulative precipitation, **(E)** average daily temperature, and **(F)** cumulative daily radiation during different growing season periods [fall (Oct-Nov), winter (Dec-Feb), jointing/anthesis (Mar-Apr), grain filling (May-mid-June)] for each YE. Boxplots portray the 5^th^ (lower whisker), 25^th^ (bottom edge), 50^th^ (solid black line), 75^th^ (top edge), and 95^th^ (upper whisker) quantiles, and mean (white diamond). On panel c, boxplots across different target plant density groups with the same letter are not statistically different (α = 0.05). On all panels, individual observations were either displayed (panel **A**), or summarized in the form of kernel density (panel b) or boxplots (panels **C–F**).

Overall across the nine field studies and 11 wheat genotypes, PD ranged from 57 to 512 plants m^-2^, and grain yield ranged from 1.1 to 8 Mg ha^-1^. The average grain yield and total number of observations was 2.7, 5.2, and 6.6 Mg ha^-1^ with 407, 517, and 236 observations for the low, medium, and high YEs, respectively (**Figures 2A, B**). Different genotypes were classified as low and high TP based on average heads plant^-1^ for each genotype. The average heads plant^-1^ was 3.4 and 4.2, and total number of observations was 723 and 437 for the low and high TP, respectively.

The ratio between achieved and target PD was greatest at the lowest target PD, and significantly decreased similarly at all YEs as target PD increased (**Figure 2C**). We also investigated whether weather pattern and variability promoted different YE, but interestingly, weather variables varied as a function of period within the growing season, but were not statistically different across YEs (**Figures 2D–F**).

### Yield Response to Density as a Function of Yield Environment and Tillering Potential

**Figure 3:**
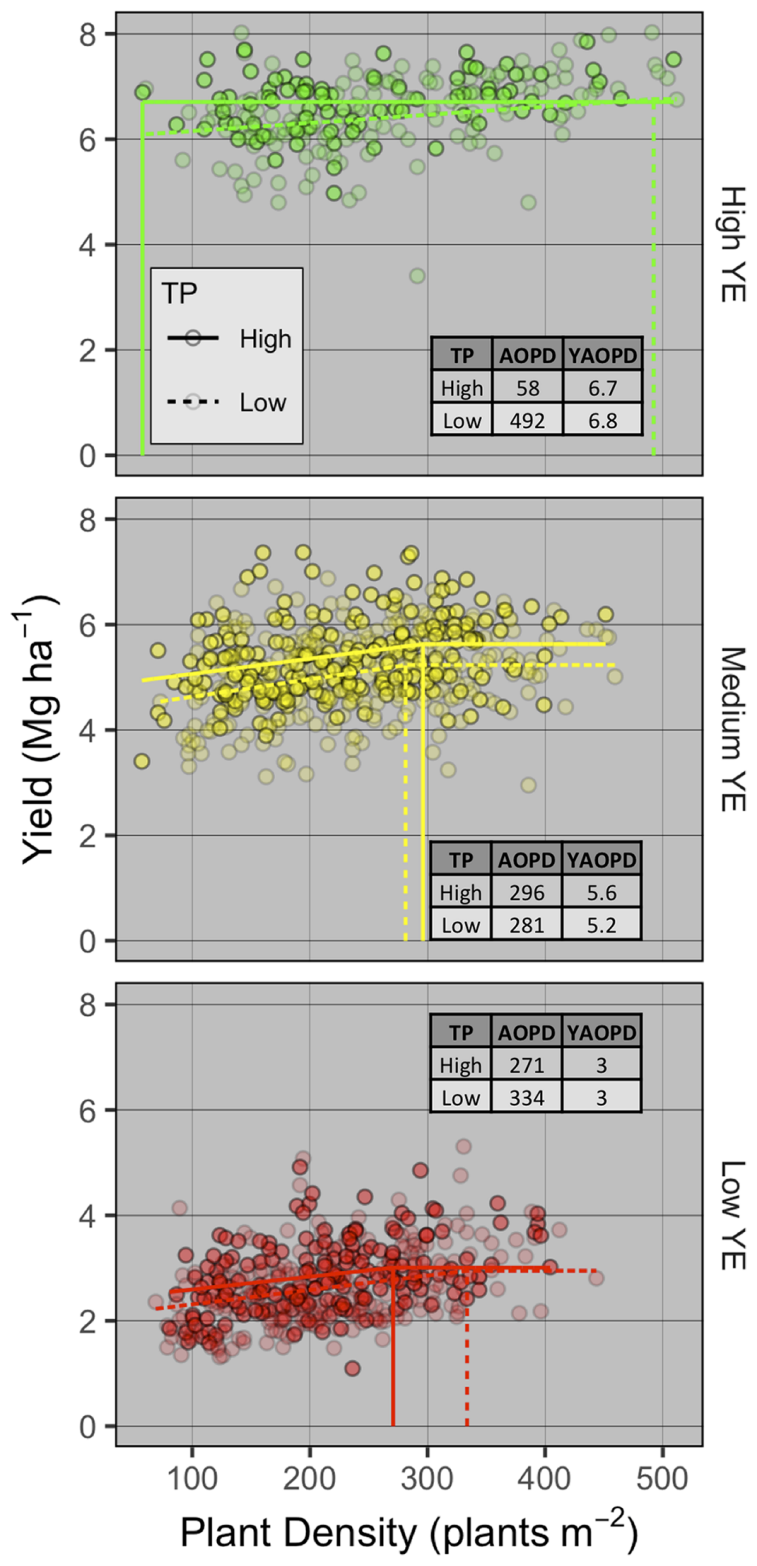
Winter wheat grain yield response to plant density and agronomic optimum plant density (AOPD) determination for different tillering potential (TP) groups (high as solid points and lines, low as transparent points and dashed lines) within the high (top), medium (intermediate), and low (bottom panel) yield environments (YE). Dashed lines are the AOPD estimates projected on the x-axis and YAOPD refers to the yield reached at the AOPD.

To understand the effect of genotype and environment on grain yield response to PD, AOPD was estimated for each combination of YE and TP (**Figure 3**). The linear-plateau model had the best fit for all YE × TP combinations, except for high-YE high-TP where grain yield did not respond to PD. At the low YE, AOPD was higher for low-TP compared to high-TP (334 vs. 271 plants m^-2^), while yield at AOPD (YAOPD) was the same for both TPs. At the medium YE, AOPD was similar between high- and low-TP (296 vs. 281 plants m^-2^, respectively). This small difference in AOPD translated into a greater difference in YAOPD of 5.6 and 5.2 Mg ha^-1^ for the high and low TPs, respectively. At the high YE, a distinct and opposite response of grain yield to PD was observed for different TPs. While grain yield at high-TP did not respond to increasing plant densities and AOPD was estimated at 58 plants m^-2^, grain yield at low-TP increased until the estimated AOPD of 492 plants m^-2^. In spite of the large difference in AOPD between both TPs, YAOPD only varied slightly (6.7 vs. 6.8 Mg ha^-1^ for the high and low TP, respectively). Despite a significant increase in grain yield from the lowest to the highest population for the low TP genotypes, the increase was only 0.7 Mg ha^-1^ and seeking the highest yield might not be economical. These results expand those found in the synthesis analysis, and demonstrate that AOPD is not only a function of management (i.e., PD) and environment (i.e., YE), but also of genotype (i.e., TP).

### Yield Components and Their Responses to Plant Density and Tiller Potential

**Figure 4:**
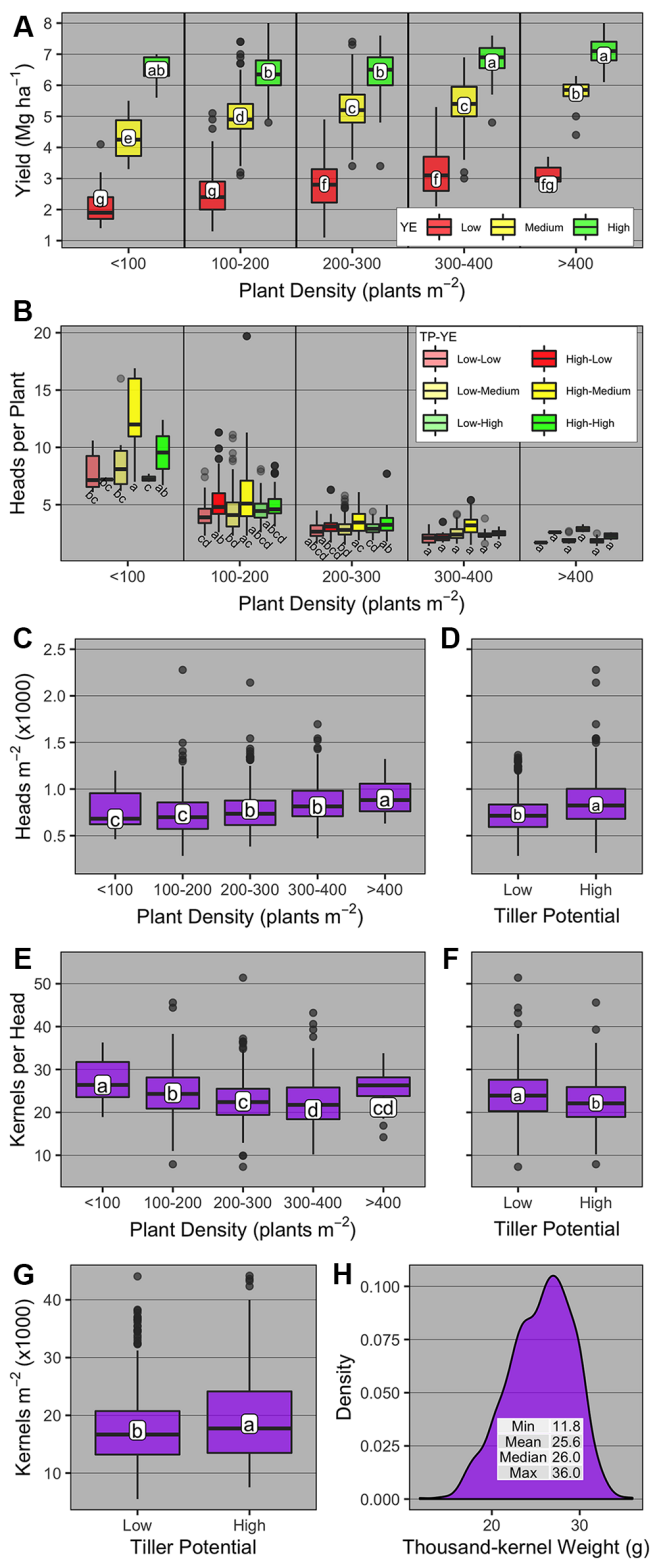
Boxplots of **(A)** winter wheat grain yield and yield components [**(B)** heads per plant; **(C, D)** heads m^-2^; **(E, F)** kernels head^-1^; **(G)** kernels m^-2^; and **(H)** thousand-kernel weight] as affected by plant density group (< 100, 100-200, 200-300, 300-400, and >400 plants m^-2^), yield environment (YE; high, medium, and low), and tillering potential (TP; high and low). On panel b, boxplots within the same plant density group with the same letter are not statistically different (α = 0.05). On all other panels, boxplots with the same letter are not statistically different across all levels shown in the panel (α = 0.05). Boxplots portray the 5^th^ (lower whisker), 25^th^ (bottom edge), 50^th^ (solid black line), 75^th^ (top edge), and 95^th^ (upper whisker) quantiles.

Winter wheat grain yield was significantly affected by PD group, YE, TP, and YE × PD group (**Supplementary Table 3**, **Figure 4**). Averaged across YE and PD group, high-TP yielded more than low-TP (4.9 vs. 4.7 Mg ha^-1^, respectively). Averaged across TP, grain yield levels had little overlap for all PD groups across different YEs (**Figure 4A**). The highest grain yields were observed at the PD groups >200 (2.8 to 2.9 Mg ha^-1^) for the low YE, > 400 (5.7 Mg ha^-1^) for the medium YE, and <100 and >300 (6.5 to 7 Mg ha^-1^) for the high YE. For the latter, the wide range of PD able to promote high yield levels is noteworthy, and demonstrates the plasticity of winter wheat plants under near non-limiting growing conditions.

The number of heads per plant was significantly affected by PD group, TP, YE × PD group, TP × PD group, and YE × TP × PD group (**Supplementary Table 3**). Overall, heads per plant was greatest at the lowest PD group and decreased thereafter for all YE × TP combinations (**Figure 4B**). At the <100 PD group, heads per plant was greatest under medium-YE high-TP and high-YE high-TP (12 and 9.6 heads per plant, respectively). For medium- and high-YE, high-TP had more heads per plant than low-TP at the <100 PD group. At the 100–200 PD group, the numerically greatest number of heads per plant was observed under medium-YE high-TP (5.4 heads per plant). The main differences in this PD group were observed at low- and medium-YE, where high-TP had significantly greater number of heads per plant than low-TP.

The number of heads m^-2^ was significantly affected by PD group and TP (**Supplementary Table 3**, **Figures 4C, D**). Averaged across YE and TP, heads m^-2^ increased from 680 to 893 as PD group increased from <100 to >400, respectively. Averaged across YE and PD group, heads m^-2^ was greater for high-TP compared to low-TP (831 vs. 729 heads m^-2^, respectively).

The number of kernels per head was significantly affected by PD group and TP (**Supplementary Table 3**, **Figures 4E, F**). Averaged across YE and TP, kernels per head was greatest at the <100 PD group (c.a., 26.4), and lowest when PD group >300 (c.a., 20.7 to 21.2). Averaged across YE and PD group, kernels per head was greatest for low-TP compared to high-TP (c.a., 24 vs. 22.2). The number of kernels m^-2^ was significantly affected only by TP (**Supplementary Table 3**), being greater for high-TP compared to low-TP (18,530 vs. 17,411 kernels m^-2^, respectively, **Figure 4G**). The thousand-kernel weight was not significantly affected by any of the explanatory variables (**Supplementary Table 3**) and varied from 11.8 to 36 g (**Figure 4H**).

The random effects analyses to understand the contribution of yield components to winter wheat grain yield suggested that at the plant level (model 1, orthogonal partition of yield components) the total yield variance contribution was in the order thousand-kernel weight > kernels per head > PD >> heads per plant, with a residual variance of 46% (**Table 3**). At the canopy level, model 2 (yield components defining final grain yield) suggested that kernels m^-2^ and thousand-kernel weight explained 37 and 23% of total yield variance, respectively, while 40% remained in the residual variance (**Table 3**). Again at the canopy level, model 3 suggested that the contributions of harvest index and aboveground biomass to total grain yield variance explained 44 and 35% of the yield variance, respectively, with a residual variance of 21% (**Table 3**).

**Table 3:** Winter wheat grain yield total variance partitioning based on different yield components models.

Model	Source of variation	Variance proportion
1	Thousand-kernel weight (g)	0.218
	Kernels per head	0.212
	Plant density (plants m^-2^)	0.111
	Heads per plant	0.001
	Residual	0.458
2	Kernels m^-2^	0.370
	Thousand-kernel weight (g)	0.229
	Residual	0.401
3	Harvest index	0.439
	Biomass (g m^-2^)	0.350
	Residual	0.211

### Yield At AOPD as a Function of Yield Environment and Tillering Potential

**Figure 5:**
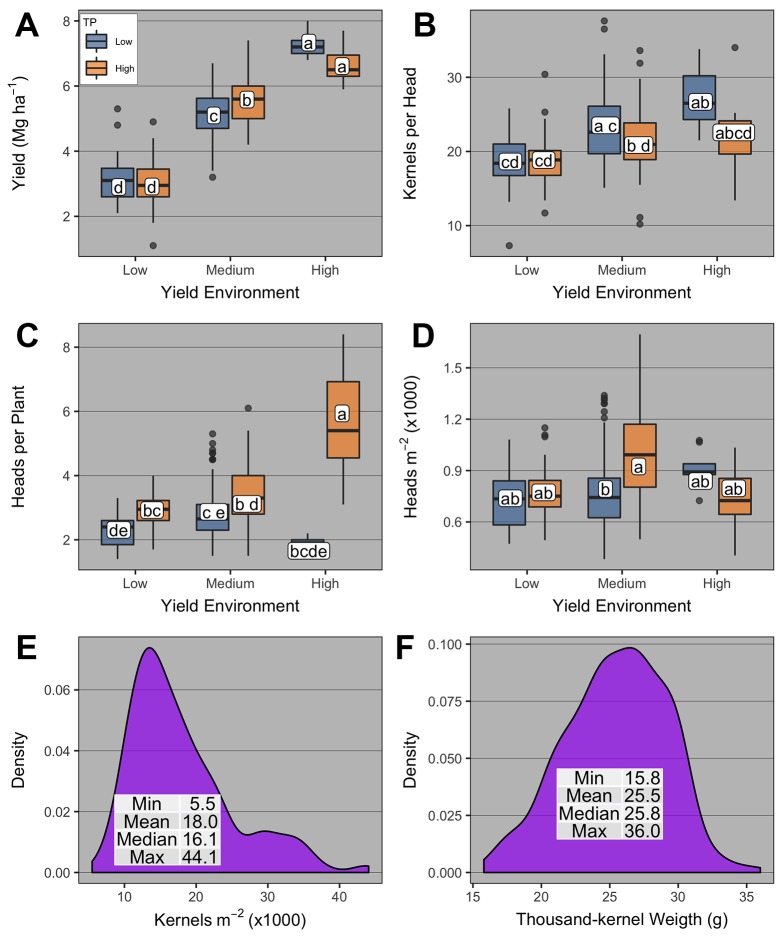
Boxplots of **(A)** winter wheat grain yield and yield components [**(B)** kernels head^-1^; **(C)** heads per plant; **(D)** heads m^-2^; **(E)** kernels m^-2^; and **(F)** thousand-kernel weight] at the agronomic optimum plant density (AOPD) as affected by yield environment (high, medium, and low), and tillering potential (TP; high and low). Boxplots with the same letter are not statistically different across all levels shown in the panel (α = 0.05). Boxplots portray the 5^th^ (lower whisker), 25^th^ (bottom edge), 50^th^ (solid black line), 75^th^ (top edge), and 95^th^ (upper whisker) quantile.

Given that AOPD was variable across different YE × TP combinations, yield and yield components at AOPD were further analyzed as a function of YE and TP (**Figure 5**). Grain yield at AOPD was significantly affected by YE and YE × TP (**Supplementary Table 4**). Grain yield varied across YEs in the order high > medium > low YE, and high-TP yielded more than low-TP only in the medium YE (5.6 vs. 5.1 Mg ha^-1^, respectively, **Figure 5A**).

The number of kernels per head was significantly affected by TP and YE × TP (**Supplementary Table 4**). Kernels per head was numerically greatest under low-TP high-YE (26.7), yet not different from other TPs at the medium- and high-YE (**Figure 5B**). Kernels per head within the high-TP were not different across YEs, while low-TP at the high-YE was significantly greater than low-TP at low-YE.

The number of heads per plant was significantly affected by TP and YE × TP (**Supplementary Table 4**). Heads per plant was greatest at high-YE high-TP (5.9) and lowest at high-YE low-TP (1.7, **Figure 5C**). High-TP had significantly higher heads per plant than low-TP in all YEs. At the canopy-scale, the number of heads m^-2^ was significantly affected by YE × TP (**Supplementary Table 4**, **Figure 5D**). The greatest number of heads m^-2^ was observed under medium-YE high-TP (924), being only different from that under medium-YE low-TP (792). The number of kernels m^-2^ and thousand-kernel weight were not affected by YE or TP (**Supplementary Table 4**) and ranged from 5.5 to 44 kernels m^-2^; and from 16 to 36 g, respectively (**Figures 5E, F**).

## Discussion

To the extent of our knowledge, this paper is the first effort on synthesizing literature data on winter wheat yield and its response to PD. Linear-plateau relationships were adjusted for the 1^st^, 50^th^, and 99^th^ quantiles, differing in the slopes of the linear models and in the breakpoint to maximize yields, demonstrating the ability of wheat to capture resources differently based on environmental potential. Yield response to PD in wheat is largely driven by the competition for resources with neighboring plants (Satorre, 1988). The literature review demonstrated that when the environment is less limited in resources (high-yielding, plateau at ~8 Mg ha^-1^), the number of plants required to maximize yield was lower relative to the medium (plateau at ~6 Mg ha^-1^) and low (plateau at ~2 Mg ha^-1^) yielding environments because plants utilized the resources more efficiently. We purposely limited our literature review and field experiments to winter wheat. While the results shown in this paper apply strictly to winter wheat, we also performed a literature review including spring wheat papers, which resulted in similar findings (i.e., yield plateau at 153 and 269 plants m^-2^ for HY and LY, *n* = 35 manuscripts; data not shown). Likewise, a recent study from Fischer et al. (2019) discussed the remarkable insensitivity of spring wheat yield response to PD in low latitude with ample resources. Thus, while these results might mostly apply to winter cereals (i.e., wheat, triticale), there is also some evidence that these results might also apply to spring wheat in certain growing conditions. However, we note that spring wheat grown in high latitudes or under lower resource availability, where the crop cycle and tillering potential might be limited by the number of accumulated thermal units, might warrant greater PD (Mehring, 2016). The work by Fischer et al. (2019) also portrayed the lack of data on PD below 100 plants m^-2^. From our review, only three studies presented minimum plant densities below 100 plants m^-2^ (McLeod et al., 1992; McLeod et al., 1996; Beres et al., 2016) and the AOPD for the high-yielding environment was attained with 141 plants m^-2^. The review from Fischer et al. (2019) highlighted a study from UK recorded by Whaley et al. (2000) that presented a maximum yield around 9.5 Mg ha^-1^ with an optimum of 100 plant m^-2^ when sowing at the optimal date. Likewise, Lollato et al. (2019) showed that yield contest winter wheat fields (e.g., high input and high yielding fields) were still able to attain their potential (c.a., 7.5 Mg ha^-1^) when sown at ~100 seeds m^-2^ (which would result in <100 plants m^-2^). This information confirms the main outcomes found on this first section of the review, under high-yielding and less limited resources the number of plants required to maximize yields in wheat is very low, below any commercially recommended number of plants for this crop. On the other spectrum of the frontier line (Q = 0.01), low yielding environments, a much lower efficiency and greater AOPD level was needed to sustain maximum yields. These results are likely a function of less resource availability and poorer growing conditions for those environments, potentially leading to lower tillering ability and less heads per unit area (Valério et al., 2013), and the need for increased plant densities.

Limitations of this review analysis were: i) the relatively small number of manuscripts included in the analysis (while many papers reported seeding rate by yield relationships, it was surprisingly rare for manuscripts to report emerged plants per unit area); and ii) the underpinning physiological mechanisms governing the yield to PD response in each YE cannot be properly dissected due to the lack of data on the yield components for each study synthesized on this dataset. In addition, lack of well-documented information on the ability of each genotype to produce tillers is also a major weakness for identifying the main causes for obtaining lower AOPD in less limited resource environments. Therefore, the dataset collected from field research studies performed in Kansas for characterizing the effect of genotype (tillering ability) by environment by management interactions was utilized on this study to provide a more detailed physiological response to the mechanisms that play a critical role in the compensation process at lower plant densities.

Results from the field studies showed interaction between YE and TP on AOPD (**Figure 3**). As was expected, a lower AOPD was observed at high YE as compared to medium-low YE (58 vs. 284 plants m^-2^) for the high TP genotypes. Likewise, Mehring (2016) suggested that the AOPD for hard red spring wheat decreased with increases in YE, and concluded that a high tillering genotype (‘Albany’) should be seeded at lower seeding rates than other genotypes with less TP to maximize yields. These findings are similar to those by Balla (1971). We also measured a lower ratio of achieved/target PD at higher seeding rates, which has been previously reported (Hanson and Lukach, 1992; Wiersma, 2002; Mehring, 2016). Anyhow, this was the case for all YEs. Even though the main effect of YE was non-significant in explaining achieved/target PD ratio, largely due to the high variability in the data, for all target PDs the numerical mean ratio followed the order high > medium > low YE. This pattern can be a consequence of suboptimal seedbed conditions (Hanson and Lukach, 1992), adverse weather and soil conditions (Mehring, 2016), or late sowing dates (Staggenborg et al., 2003). Thus, Dahlke et al. (1993); Staggenborg et al. (2003), and Lloveras et al. (2004) indicated the need to increase seeding rates with late sowing dates, because the shorter growing period reduced the individual plant growth and tiller production. In addition, Lloveras et al. (2004) reported a linear relationship between yield and PD in conditions of dry winter, showing the greater PD required to increase yield when water availability limited the crop growth. While this type of post-mortem analysis is useful in understanding the factors contributing to the yield-PD relationship, a producer will not know future weather to adjust seeding rate decisions accordingly. Within this context, the appropriate decision could take into account the yield history (past years of average yield from the same field) in each field to determine an expected YE. Nonetheless, weather uncertainty plays a major role in the outplay of various management practices, including seeding rate. A producer may respond to weather uncertainty by selecting conservative management practices, which in this case correspond to higher seeding rates, and/or utilizing genotypes with high TP.

Surprisingly, in our study it was not possible to characterize each YE with weather variables. The lack of significant weather effect was likely the result of low statistical power given that the total number of observations for a given weather variable and period was nine. Nonetheless, greater cumulative precipitation in the Fall, higher average temperature, and lower cumulative radiation during the Fall and Winter periods were evident when comparing low and high YEs (**Figures 2D, F**, respectively). We hypothesized i) that differences in intercepted solar radiation between YEs and among seeding rates within the same YE might have led to some of the observed differences in yield response (**Box 1**); and ii) that differences among site-years in nitrogen supply (e.g., either lower nitrogen supply due to losses, or higher nitrogen supply due to the mineralization of the soil organic matter) and/or nitrogen demand by the plants could have impacted the yield response at different YEs. This hypothesis is corroborated by observed site-year differences in exported nitrogen in the grain at different yield levels (**Supplementary Figure 1**).

Box 1 **Intercepted solar radiation as affected by seeding rate and yield environments**

**Figure 6:**
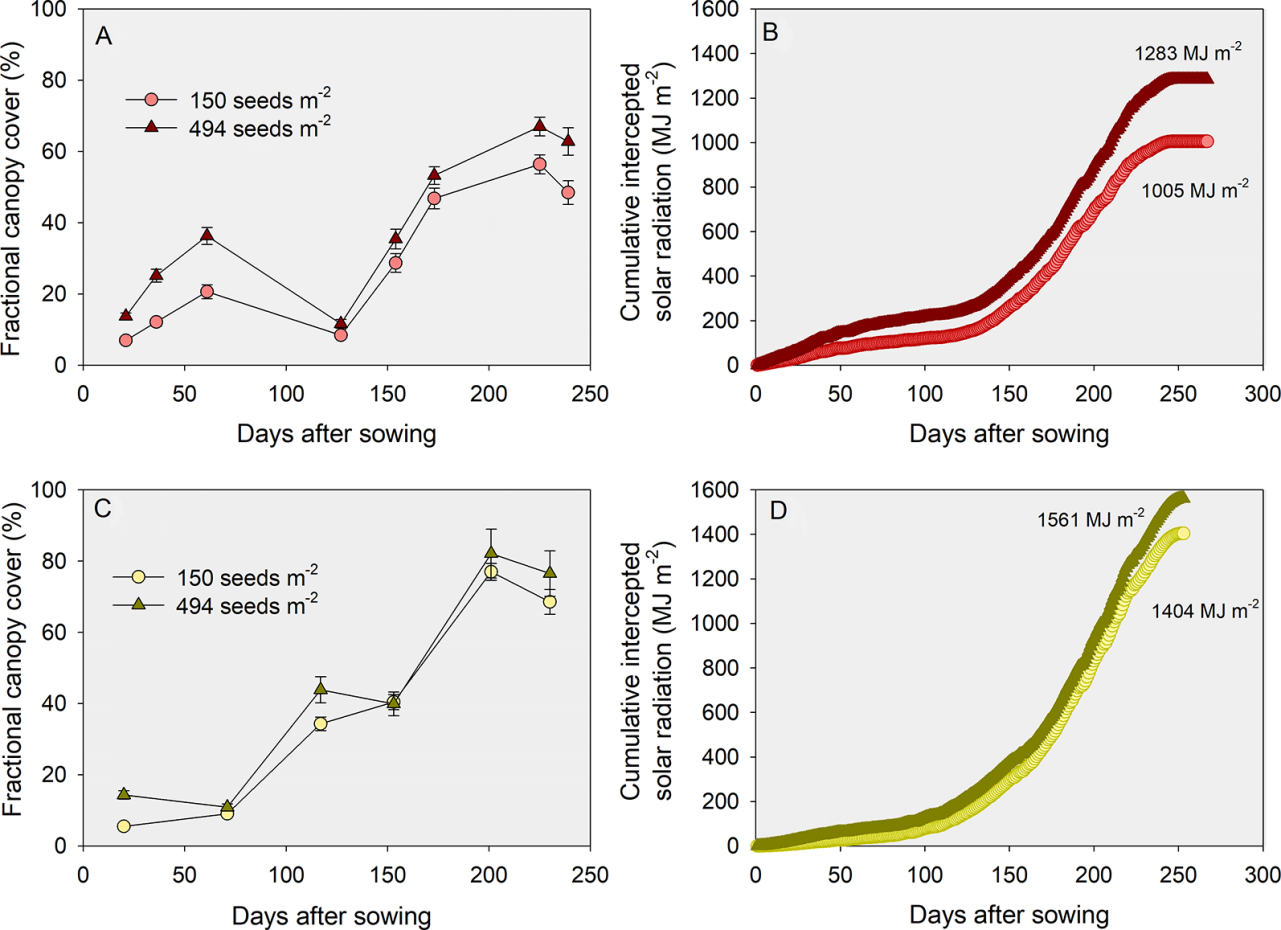
Growing season dynamics of fractional green canopy cover **(A, C)** and cumulative intercepted solar radiation during the growing season **(B, D)**, for a low **(A**, **B)** and a medium **(C**, **D)** yield environment. Data represents the low tillering potential varieties sown at the lowest (150 seeds m^-2^) or highest (494 seeds m^-2^) seeding rates.

Data on fractional green canopy cover for two YEs (low vs. medium) was collected during the 2015-16 growing season [i.e., Manhattan (average yield of 2.9 Mg ha^-1^) and Hutchinson (average yield of 5.2 Mg ha^-1^)]. The fractional green canopy cover was measured using a methodology similar to Purcell (2000), where digital photographs encompassing one meter square were taken in eight to ten different dates in the season. Photos were analyzed using Canopeo (Patrignani and Ochsner, 2015). **Figures 6A, C** portray the growing season dynamics of fractional green canopy cover, calculated by assuming a linear increase or decrease between consecutive measurements. Incident solar radiation measured in a nearby weather station was multiplied by each respective daily fractional green cover, and a cumulative value of intercepted solar radiation for the growing season was calculated (**Figures 6B, D**).

This exploratory analysis showed that: i) fractional green canopy cover was as high as 82% in the medium YE but never surpassed 67% in the low YE; ii) cumulative intercepted solar radiation was considerably lower in the low relative to the medium YE (c. 1145 vs. 1465 MJ m^-2^) despite similar growing season total (3710 vs. 3571 MJ m^-2^, respectively); iii) differences in cumulative intercepted solar radiation between the lowest and the highest seeding rate were greater in the low (c. 1005 vs. 1283 MJ m^-2^, or 28%) relative to the medium (1404 vs. 1562 MJ m^-2^, or 11%) YE, and iv) differences in intercepted solar radiation between the low versus high TP varieties were negligible regardless of the environments and seeding rates (data not shown). Considering a radiation use efficiency of 1.4 g MJ^-1^ and a harvest index of 0.4 (Lollato and Edwards, 2015), differences in intercepted solar radiation led to differences in yield potential of 6.4 to 8.2 Mg ha^-1^ between YEs. The difference in yield potential between seeding rates was expectedly greater at the low (7.2 versus 5.6 Mg ha^-1^ for 494 and 150 seeds m^-2^, respectively) relative to the medium YE (8.7 vs. 7.9 Mg ha^-1^, respectively). We note in passing that the decrease in percent canopy cover measured in **Figure 6A** between days after sowing 61 (c.a., 19–33%) and 127 (c.a., 9 and 11%) resulted from losses in green leaf area due to cold winter temperatures coupled with no-till and large amounts of maize residue. This loss in canopy area was not measured in the warmer winter and conventional tillage practices in the southern location (**Figure 6C**).

A genotypic effect was observed on the AOPD, here studied by classifying the genotypes on TP groups. These results are supported by research suggesting a significant genotype by seeding rate interaction (Pendleton and Dungan, 1960; Briggs and Ayten-Fisu, 1979; Faris and De Pauw, 1980; Baker, 1982; Anderson and Barclay, 1991; Wiersma, 2002; Mehring, 2016). The field research dataset confirmed the influence of TP, more specifically in both low (~3 Mg ha^-1^) and high (~6 Mg ha^-1^) YEs. Clearly, for low YE, genotypes that had a greater TP resulted in a reduced AOPD by 23% relative to those classified as lower TP (**Figure 3**), while no difference between TPs were observed in the medium YE. Surprisingly, the AOPD at high YE changed with the TP. Similarly, Valério et al. (2009) reported greater seeding rates needed to obtain maximum yield for low TP genotypes, compared to high TP (417 to 555 vs. 221 to 422 seeds m^-2^, respectively), with similar findings by Mehring (2016) and Balla (1971). Moreover, for the low TP genotypes, these authors observed linear relationships between yield and seeding rate with low yields, but the relationship was mostly quadratic in site-years with greater yields. This reinforces the idea of increasing seeding rates/PDs as the environment is more restrictive for wheat growth. In spite of numerical differences, the AOPD levels observed on the field studies followed a similar trend to those found on the synthesis analysis, with a decrease in AOPD as YE changes from low to high, except for the high YE and low TP condition. The studies included in the synthesis analysis did not report on genotype TP, and thus our results cannot be validated with the same literature dataset. These differences in AOPD, especially at high YE, are significant for seeding rate decisions, and future yield-PD studies should also report information on genotypic TP. Furthermore, AOPD levels observed from the field studies for the low and medium YEs are similar to the first- (246 seeds m^-2^) and third-quantile (304 seeds m^-2^) of the seeding rate distribution of 100 intensively-managed wheat fields surveyed through the Kansas Wheat Yield Contest from 2010 through 2017 (Lollato et al., 2019). The authors found that in high YEs (i.e. 0.99 quantile), seeding rates greater than 305 seeds m^-2^ were negatively correlated with yield (loss of 2.7 Mg ha^-1^ for each 100-seed m^-2^ increment above 305 seeds m^-2^). This behavior was not observed in our field studies high YE analysis, where greater seeding rates were optimal for low TP; and for high TP, while not optimal, high seeding rates were neither detrimental.

Given the YE-TP specific relationship of decreasing PD with increasing seeding rates, under the high-YE low-TP condition it would be required 686 seeds m^-2^ to achieve a PD of 492 plants m^-2^ (i.e., AOPD for this case). Assuming this seeding rate, a seed cost of US$1.54 per 100,000 seeds, a yield at AOPD of 6.8 Mg ha^-1^, and a grain price of US$157 Mg^-1^, the marginal profit for the high-YE low-TP condition when seeded to match AOPD is US$963 ha^-1^, compared to US$949 ha^-1^ if seeded at a low PD of 60 seeds m^-2^ with a yield 6.1 Mg ha^-1^. Thus, in spite of a large difference in seeding rate, the high-YE low-TP condition would still economically benefit from a seeding rate to match its AOPD of 492 plants m^-2^. The difference in AOPD between the low and high TP genotypes can be used to anticipate the economics between conventional vs. hybrid wheat. Hybrids usually have a greater tillering potential as well as grain yield than conventional wheat (Rai et al., 1970; Curtis et al., 2002). Thus, for each YE, we assumed hybrid AOPD to be the same as that from high TP, and hybrid yield at AOPD to be 10% greater than yield at AOPD from high TP. Those numbers were compared against AOPD and yield at AOPD from low TP (representing conventional wheat). Further assuming a seed cost of US$1.54 and US$3.85 [i.e., 2.5x greater for hybrid, Curtis et al. (2002)] per 100,000 seeds for conventional and hybrid wheat, respectively, and a grain price of US$157 Mg^-1^, the marginal profit difference between hybrid and conventional wheat would be -30, 44, and 171 US$ ha^-1^ for the low, medium, and high YEs, respectively. Thus, under all the stated assumptions, the economic advantage of using hybrid over conventional wheat would be warranted under responsive environments, and discouraged under limiting environments.

In our research, the statistical models that usually maximized fit when representing wheat yield as affected by PD were linear-plateau. While the literature reports a wide range of models representing wheat yield as function of seeding rate (e.g., linear, quadratic, quadratic-plateau and lack of response; Whaley et al., 2000; Lloveras et al., 2004; Fischer et al., 2019), the quadratic is usually the most often reported to represent lodging and other potential yield losses due to increased pressure of insects and diseases at high populations and/or high yielding conditions (Lollato and Edwards, 2015; Mehring, 2016). In our study, diseases and insects were not a confounding factor due to prophylactic application of pesticides. Likewise, while lodging is recognized as a reoccurring issue in wheat grown under high populations (Holliday, 1960; Kirby, 1967; Faris and De Pauw, 1980; Orloff, 2014; Mehring, 2016), it ranged from non-existing to moderate in most of the site-years studied, not impacting yields (data not shown). Nonetheless, while this research grouped genotypes based on their expressed TP, future research should also investigate the effects of straw strength (Mehring, 2016), maturity (Hucl and Baker, 1988), and, when not controlled, disease and insect reactions of the studied genotypes.

Our findings related to the differential yield-PD response under varying YEs and TPs can be potentially used to guide variable seeding rate efforts. As an example, a producer could split his/her fields and sub-field regions into low-medium vs. high YEs based on historical yield trends. Then, low-medium YEs could be seeded at rates ranging from 300–350 seeds m^-2^ with the option of using high TP genotypes for greater plasticity in yield response. Moreover, high YEs could be seeded either at a low (< 100 seeds m^-2^) or high (~500 seeds m^-2^) rates, depending on the genotype TP, respectively. Nonetheless, this approach assumes that i) YEs remain stable through time (e.g. high YEs are high-yielding regardless of the year); ii) the yield limiting factors under low-medium YE are stationary regarding what caused them to be lower-yielding in the first place (e.g. soil texture, slope, etc.); and iii) the seed cost-to-grain price ratio remains stable. Independently of these assumptions being met, a wheat variable seeding rate technology should be site-specifically validated in order to optimize profitability.

Wheat genotypes differed in their plasticity to compensate for variations in PD by modifying different yield components, including the number of productive tillers (Lloveras et al., 2004). Wheat TP is a quantitative trait (Li et al., 2002) and thus genotypic differences in TP among wheat genotypes exist (Hucl and Baker, 1988; Anderson and Barclay, 1991; Mehring, 2016) and its expression depends on environmental conditions such as precipitation (Anderson and Barclay, 1991) and the genotypes' length of vegetative period (Hucl and Baker, 1988). Then, seems likely that under restrictive growing conditions (low YE), genotypes with a greater TP allowed to reach the maximum yield with a lower number of plants per unit area, while low TP genotypes were not able to fully compensate for the decreased number of plants by increasing the number of tillers per plant. Therefore, genotypes with lower TP are more dependent on seeding rate/PD for maximizing yield (Geleta et al., 2002; Valério et al., 2013).

Variations in yield components could explain the effect of YE and TP on AOPD. Our results showed that tillers and consequently heads per plant increased with reductions in PD, but overall, the increment was greater for the high TP genotypes compared to the low ones (**Figure 4B**). Consequently, the high TP genotypes could compensate the reductions in plants with more tillers and head per plant, avoiding a great reduction in heads number per unit area. Thus, a lower reduction in heads per unit area due to low PD occurred at high YE compared to low YE. Similarly, Mehring (2016) suggested that higher tillering genotypes had more tillers per plant than lower tillering genotypes at the two lowest out of five seeding rates evaluated. Wheat crops growing at low PD increased green area per plant and the duration of tiller formation (Whaley et al., 2000), which explains why yield did not decrease proportionally to PD variations. A reduction in PD decreased the number of heads per unit area but increased the number of kernels per head. However, some differences in these mechanisms were observed between TP groups. For the low TP, the increase in kernels per head was not enough to compensate the reduction in heads per unit area, and consequently, the number of kernels per unit area was reduced, negatively affecting the yield. In agreement, Arduini et al. (2006) observed an increase in the number of kernels per head with the decrease of seeding rate, but this yield gain was not enough to fully compensate for the lower number of heads per unit area. Moreover, Arduini et al. (2006) and Slafer et al. (2014) reported how yield is regulated by yield components and environment, and stated that number of kernels per area is a coarse and seed weight is a fine regulator of wheat yield. While AOPD estimation prioritizes grain yield, grain quality parameters are an important consideration when evaluating yield-PD responses. In the current research, the relationships between PD and grain protein concentration, test weight, and thousand-kernel weight were genotype-dependent (**Box 2**).

Box 2 **Grain quality parameters as affected by plant density and genotypes**

The effect of PD on grain quality was assessed by evaluating the relationships between: i) grain protein concentration and PD as a function of genotype; iii) test weight and PD as function of genotype; and iv) TKW and PD as a function of genotype. For each analysis, a range of models with different covariates (site-year, YE, genotype, TP) main and interacting effects were evaluated, and the one with the lowest AIC was chosen. Models residuals were diagnosed, and when necessary, outliers (less than -3 standardized residuals) were removed, and/or within-group error variances were allowed to vary according to site-year in order to address residual variance heteroscedasticity.

**Figure 7:**
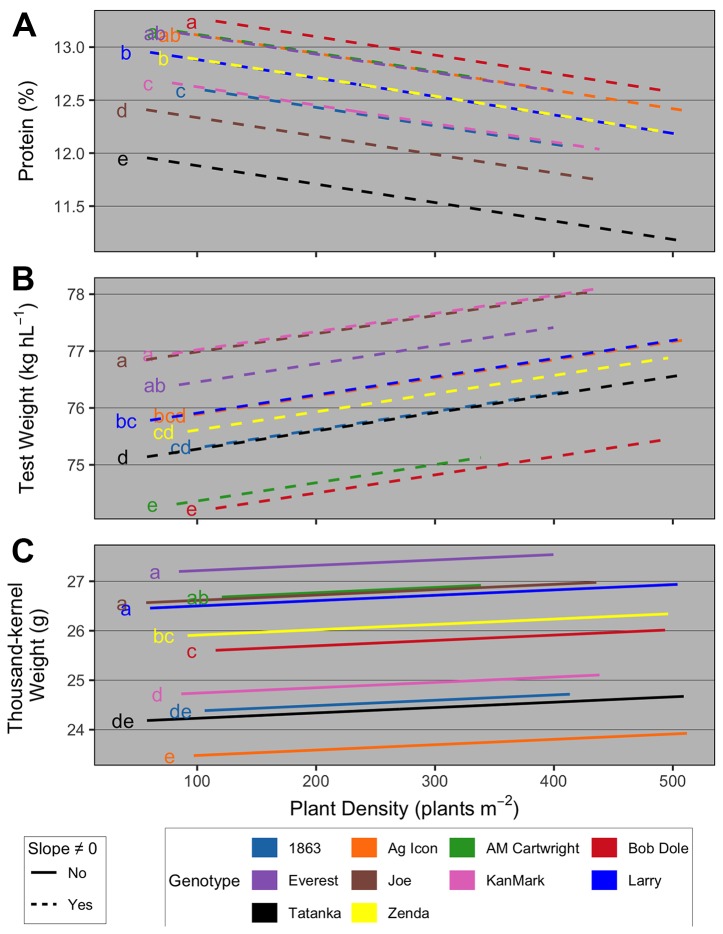
Relationship between **(A)** grain protein concentration and plant density as a function of genotype; **(B)** test weight and plant density as function of genotype; and **(C)** thousand-kernel weight and plant density as a function of genotype. Different line types represent slopes that were significantly different than zero (dashed) and not significantly different than zero (solid). At each panel, the y-intercept of regression lines followed by the same letter are not statistically different (α = 0.05).

Overall, results suggested that i) grain protein concentration decreased with increasing PD at the same rate for all genotypes, with a genotype-dependent *y*-intercept (**Figure 7A**); ii) test weight increased with increasing PD at the same rate for all genotypes, with a genotype-dependent *y*-intercept (**Figure 7B**); and iii) TKW did not vary with increasing PD, but had a genotype-dependent main effect on the *y*-intercept (**Figure 7C**). First, similarly to available literature (Otteson et al., 2008; Williams et al., 2008; Rozbicki et al., 2015), these results highlight the importance of genotype on wheat quality determination, as using actual genotype rather than its TP explained much greater proportion of the variability in quality parameters. Regarding management, our results are also supported by Roth et al. (1984) and Geleta et al. (2002), who suggested that wheat test weight increased with increases in seeding rate due to the greater proportion of primary spikes compared to secondary tillers as seeding rate increased. Greater test weight on the primary spikes compared to tillers have been reported by Gautam et al. (2012).

Our study provides a unique perspective on the AOPD needed for maximizing wheat productivity at different yield levels. The data collected from both the review and the field research studies confirmed that AOPD is lower under high-yielding and less resource limited environments. The latter has been recently reported by Fischer et al. (2019) in a high fertility, irrigated environment; concluding that the great plasticity in wheat by tillering appears to explain the lower number of plants required to maximize light interception and increase yields. Nonetheless data on TP was not reported by Fischer et al. (2019). Therefore, this study closes this unknown research gap on the need of lower AOPD for high-yielding environments by demonstrating with the field research data set that greater tillering ability from diverse wheat genotypes is a main factor for improving yields at very low plant densities.

## Conclusions

Major findings of this study were: i) the review analysis portrayed new insights of differences in AOPD at varying YEs, reducing the AOPD as the attainable yield increases (with AOPD moving from 397 plants m^-2^ for the low YE to 191 plants m^-2^ for the high YE); ii) the field dataset confirmed the trend observed in the review but expanded on the physiological mechanisms underpinning the yield response to PD for wheat, highlighting the following points: a) high TP reduces the AOPD mainly in high and low YEs, b) at canopy-scale, both final heads and kernel number were the main factors improving yield response to PD under high TP, c) under varying YEs, at per-plant-scale, a compensation between heads per plant and kernels per head were the main factors contributing to yield with different TP genotypes.

As evidenced by the synthesis-analysis and expanded by the field studies to include TP, AOPD varies as a function of YE primarily, and to a lesser extent as a function of TP, except at high YE where TP is an important modulator of yield. Based on this, a producer may select different seeding rates and genotypes with varying levels of TP depending on a given field YE and the producer risk aversion. Either increasing seeding rates or selecting high TP genotypes could be used to decrease weather-related production risk. However, the former may not be the most economical practice if adverse weather does not happen and a lower seeding rate may have produced equally well. Therefore, we demonstrated with this work aspects of management and genotype that producers can select to better match their profitability and risk potential.

## Data Availability Statement

The datasets generated for this study are available on request to the author(s).

## Author Contributions

LB and WC performed the statistical analyses and drafted the manuscript. RL and AF designed the field experiment and, aided by BJ, CR, and GZ, conducted the field experiments. LB and IC coordinated data collection and led the review synthesis analysis, and LB, RL, and IC guided statistical analysis and development of the entire manuscript. All authors contributed, reviewed and edited the final manuscript.

## Funding

We thank the Kansas Wheat Alliance for sponsoring the three years of field research conducted in Kansas. We thank the John Deere Company for providing financial support to the synthesis analysis and data analysis parts of this project.

## Conflict of Interest

Authors CF, YW, SY, and PB were employed by the company John Deere.

The remaining authors declare that the research was conducted in the absence of any commercial or financial relationships that could be construed as a potential conflict of interest.

The authors declare that this study received funding from John Deere. The funder was not involved in the study design, collection, analysis, interpretation of data, the writing of this article or the decision to submit it for publication, but provided feedback on the original research question and final version of this manuscript.

## Abbreviations

AOPD, agronomic optimum plant density; PD, plant density; TP, tillering potential; YE, yield environment.
