# Supplementary material for: Winter Wheat Yield Response to Plant Density as a Function of Yield Environment and Tillering Potential: A Review and Field Studies
Source: Front Plant Sci. 2020 Mar 5;11:54. doi: 10.3389/fpls.2020.00054 (PMC7066254; doi:10.3389/fpls.2020.00054)
Supplement: Supplementary file 1 [file DataSheet_1.docx]

Supplementary Material

# Supplementary Tables and Figures

**Supplementary Table 1**. Soil fertility measured at sowing in each site-year including pH, Mehlich-3 extractable phosphorus (P), potassium (K), calcium (Ca), magnesium (Mg), sodium (Na), ammonium nitrogen (NH_4_–N) and nitrate nitrogen (NO_3_^-^–N), chloride (Cl), sulfate sulfur (SO_4_^2-^–S), organic matter (OM) and cation exchange capacity (CEC) in the 0-15 cm depth in different locations in Kansas, USA. Abbreviations: MHT, Manhattan; HUT, Hutchinson; BEL, Belleville; ASB, Ashland Bottoms; GTB, Great Bend; HAY, Hays; and LEO, Leoti. Site-year abbreviation is followed by harvest year. na, not applicable (data not collected in the corresponding site-year).

| Site-year | pH | P | K | Ca | Mg | Na | NH_4_-N | NO_3_-N | Cl | SO_4_^2-^-S | OM | CEC |
| --- | --- | --- | --- | --- | --- | --- | --- | --- | --- | --- | --- | --- |
|  |  |  |  |  |  | mg kg ^-1^ |  |  |  |  | % | cmolc 100g ^-1^ |
| ASB18 | 5.4 | 40 | 169 | 857 | 110 | 2.8 | 5.3 | 30.6 | 4.1 | 1.7 | 1.2 | 8.9 |
| BEL17 | 4.9 | 65 | 492 | 1217 | 176 | 14.2 | 15.9 | 21.3 | 13.0 | 7.4 | 2.8 | 24.6 |
| GTB18 | 5.3 | 175 | 605 | 1719 | 430 | 23.2 | 4.6 | 40.6 | 9.1 | 7.2 | 2.7 | 26.2 |
| HUT16 | 5.1 | 80 | 229 | 1402 | 222 | 10.7 | 9.2 | 4.2 | na | na | 2.0 | na |
| HUT17 | 7.5 | 58 | 318 | 3851 | 172 | 15.3 | 17.1 | 7.1 | 3.3 | 5.4 | 2.6 | 21.6 |
| HUT8 | 6.1 | 77 | 218 | 1886 | 238 | 10.7 | 3.7 | 6.2 | 7.2 | 3.4 | 2.4 | 19.6 |
| LEO18 | 6.2 | 70 | 649 | 2131 | 386 | 13.3 | 3.6 | 13.2 | 5.1 | 4.0 | 2.3 | 23.1 |
| MAN16 | 6.6 | 40 | 210 | 4045 | 311 | 22.8 | 29.7 | 9.7 | 4.8 | 7.0 | 3.9 | 26.8 |

**Supplementary Table 2.** Average temperature (Temp) and cumulative precipitation (Precip.), grass evapotranspiration (ET_o_), and incident solar radiation (Rad) in each site-year at different locations in Kansas, USA. Abbreviations: MHT, Manhattan; HUT, Hutchinson; BEL, Belleville; ASB, Ashland Bottoms; GTB, Great Bend; HAY, Hays; and LEO, Leoti. Site-year abbreviation is followed by harvest year.

|  | Site-year | | | | | | | | |
| --- | --- | --- | --- | --- | --- | --- | --- | --- | --- |
| Season | MHT16 | HUT16 | HUT17 | HUT18 | BEL17 | ASB18 | GTB18 | HAY18 | LEO18 |
|  |  |  |  |  | Temp (°C) |  |  |  |  |
| Fall | 9.8 | 9.4 | 8.8 | 7.0 | 6.7 | 7.3 | 7.6 | 6.3 | 6.3 |
| Winter | 4.9 | 5.1 | 5.8 | 2.3 | 3.2 | 2.0 | 3.1 | 1.9 | 2.3 |
| Spring | 19.6 | 18.8 | 18.6 | 18.8 | 17.4 | 19.0 | 19.3 | 18.1 | 17.3 |
|  |  |  |  |  | Precip. (mm) |  |  |  |  |
| Fall | 202 | 184 | 47 | 52 | 91 | 98 | 73 | 53 | 44 |
| Winter | 32 | 55 | 149 | 60 | 66 | 38 | 40 | 11 | 5 |
| Spring | 349 | 384 | 295 | 264 | 247 | 181 | 313 | 204 | 158 |
|  |  |  |  |  | ET_o_ (mm) |  |  |  |  |
| Fall | 200 | 217 | 226 | 232 | 168 | 200 | 203 | 247 | 264 |
| Winter | 217 | 231 | 233 | 220 | 182 | 182 | 199 | 243 | 254 |
| Spring | 460 | 451 | 454 | 522 | 456 | 476 | 476 | 569 | 588 |
|  |  |  |  | Rad (MJ m^-2^) | | |  |  |  |
| Fall | 870 | 928 | 950 | 876 | 878 | 961 | 967 | 943 | 910 |
| Winter | 1043 | 1158 | 1026 | 1065 | 994 | 1118 | 1197 | 1150 | 1155 |
| Spring | 1908 | 1913 | 1863 | 1971 | 2003 | 1973 | 2102 | 2041 | 2020 |

**Supplementary Table 3**. Analysis of variance for winter wheat grain yield and yield components as a function of plant density group (PDG), yield environment (YE), tillering potential (TP), and their interactions. Numbers inside table are p-values.

| Source of Variation | Grain Yield | Heads per plant | Heads m^-2^ | Kernels per head | Kernels m^-^² | Thousand-kernel weight |
| --- | --- | --- | --- | --- | --- | --- |
| PDG | <0.001 | <0.001 | <0.001 | <0.001 | 0.063 | 0.267 |
| YE | <0.001 | 0.659 | 0.890 | 0.363 | 0.619 | 0.150 |
| TP | 0.003 | <0.001 | <0.001 | 0.002 | 0.049 | 0.761 |
| YE × PDG | 0.026 | 0.017 | 0.052 | 0.367 | 0.174 | 0.598 |
| TP × PDG | 0.380 | 0.007 | 0.613 | 0.946 | 0.694 | 0.501 |
| YE × TP | 0.084 | 0.068 | 0.342 | 0.877 | 0.136 | 0.826 |
| YE × TP × PDG | 0.473 | 0.022 | 0.842 | 0.730 | 0.720 | 0.773 |

**Supplementary Table 4**. Analysis of variance for winter wheat grain yield and yield components at the agronomic optimum plant density as a function of yield environment (YE), tillering potential (TP), and their interaction. Numbers inside table are p-values.

| Source of Variation | Grain Yield | Kernels per head | Heads per plant | Heads m^-^² | Kernels m^-^² | Thousand-kernel weight |
| --- | --- | --- | --- | --- | --- | --- |
| YE | <0.001 | 0.089 | 0.188 | 0.826 | 0.581 | 0.384 |
| TP | 0.620 | 0.011 | <0.001 | 0.299 | 0.318 | 0.494 |
| YE × TP | <0.001 | 0.008 | <0.001 | 0.029 | 0.309 | 0.271 |

**
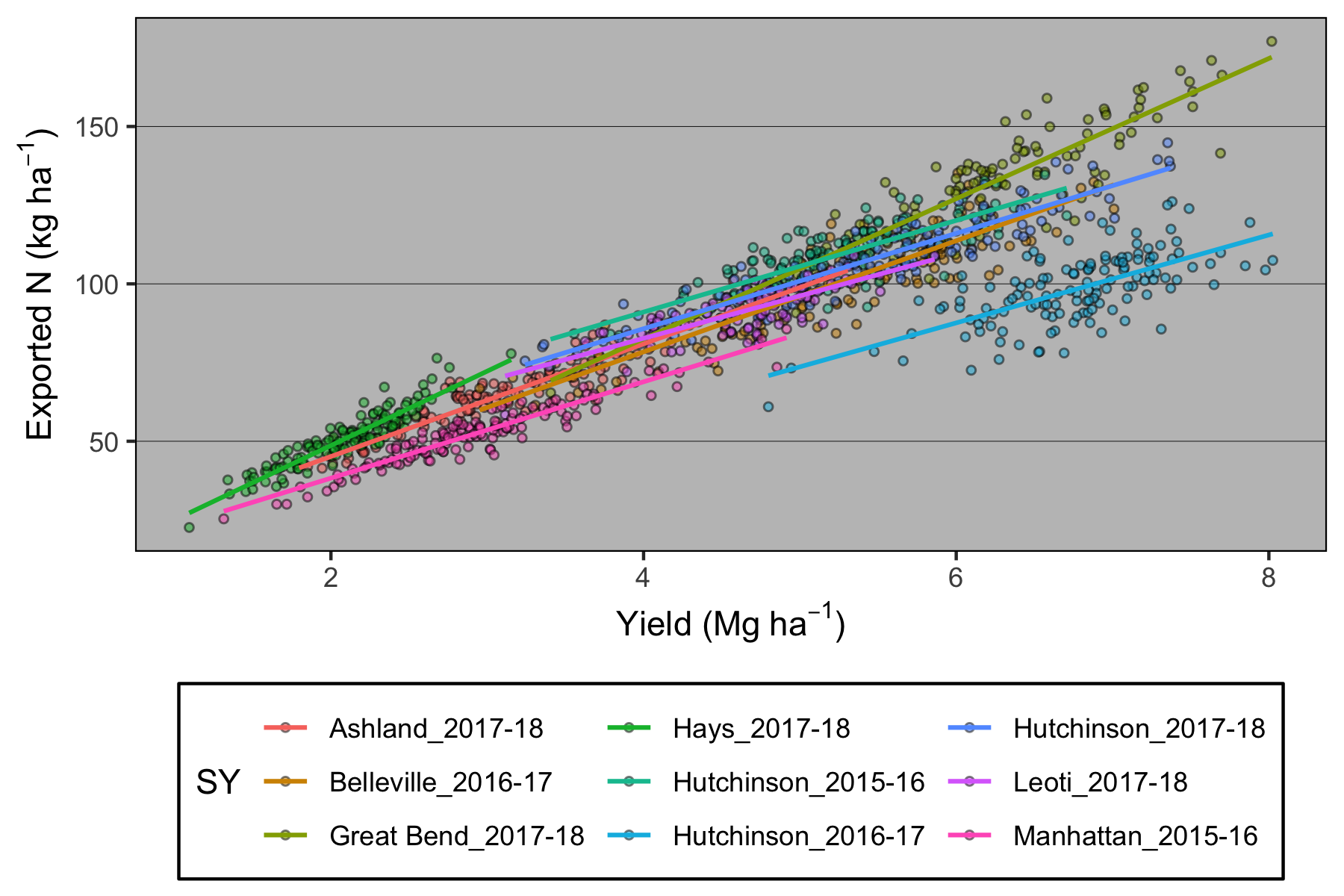
**

Supplementary Figure 1. Grain exported nitrogen response to grain yield as a function of site-year (SY).
